# Real driving cycles and emissions for urban freight transport

**DOI:** 10.3389/fdata.2024.1375455

**Published:** 2024-07-08

**Authors:** Julie Anne Holanda Azevedo, Demostenis Ramos Cassiano, Bruno Vieira Bertoncini

**Affiliations:** ^1^Rural Development Institute, University for International Integration of the Afro-Brazilian Lusophony, Redenção, Ceará, Brazil; ^2^Department of Transport Engineering, Federal University of Ceará, Fortaleza, Ceará, Brazil

**Keywords:** driving cycles, emissions pollutants, land use, freight transport, PEMS - portable emissions measurement system

## Abstract

This paper aims to evaluate the driving style effects, through the construction of driving cycles, on the polluting gases, in the context of urban freight transportation. For this, the method used was the construction of cycles through the Vehicle Specific Power (VSP) parameter, which considers instantaneous vehicle and road parameters better to represent driving patterns and freight transportation's environmental impacts. The study was conducted in Fortaleza city, Ceará, Brazil, with a professional driver's group. The road types, land use and traffic light location were considered to analyze and discuss the results. The results show collector roads presented higher speeds than arterial roads, and the use of the land around the road also directly impacted vehicle driving patterns. Regarding CO_2_ emissions, higher concentrations measured were observed on the arterial roads.

## 1 Introduction

Cities manifest strong dependence on freight transport systems to efficiently ensure the flow of goods and services and the availability of resources needed to maintain the economy and quality of life (Marcucci and Gatta, 2014; Holguin-Veras et al., 2021). Organizing in urban areas is an efficient way to exchange goods and services; as Holguin-Veras et al. (2021) point out, most people and economic activities use, at some level, goods that are traded and, consequently, have been moved. Thus, logistics plays a crucial role in managing the entire supply chain. Logistics operations, although relevant, bring externalities (McKinnon et al., 2015; Martí et al., 2017; Cassiano et al., 2021). In this context, incorporating freight transport as an integral agent in urban planning is vital, particularly in understanding the relationship between freight and aspects of urban forms, such as land use, occupancy, and urban density (Silva and Marins, 2019; Holguin-Veras et al., 2021). Silva and Marins (2019) state that high urban density may imply higher demand for goods and consequently increased trips for goods distribution. McLeod et al. (2019) point out that in cities, freight movements are much more complex than passenger transportation since it involves interactions between companies at different stages of supply chains. Thus, those who should plan urban transport policies usually lack a detailed view of the complex factors that influence urban freight transport (UFT).

As a result of the effort to understand the UFT as a complex phenomenon, Cassiano et al. (2021) proposed a conceptual model that represents the last mile operation in urban areas focusing on impact measures capable of guiding decision-makers to a sustainable reality (Shen et al., 2015; Achour and Olabi, 2016; Byun and Choi, 2021). The model is based on the relationship between the Urban Activities Subsystem (UAS) and the Transportation Subsystem (TS) and how this relationship impacts the UFT operation and, consequently, the stakeholders. Practically, the use of the proposed model has as the main gain the vision and development of projects in a holistic way. Initially, the focus on stakeholders allows the UFT planning process to address all its local specificities, thus allowing the proposition of ideas that cover the different objectives and existing needs. With this, the model presented by Cassiano et al. (2021) abandons the classic notion, in which the urban environment is summarized to the space in which the process of generating freight trips occurs and starts to approach the UFT systematically, i.e., the buying process starts in the UAS, moves to the TS with the generation of the trip and reaches the destination. In this way, it is possible to approach from classical supply and demand relationships to consumer behavioral studies and thus produce indicators or impact measures that feed from classical models to models that focus on cause-and-effect relationships, for example, studying how the relationship between UFT operation and urban form affects air quality in urban centers (Prefeitura Municipal de Fortaleza, 2012). In this regard, freight demand models stand as fundamental since according to Comi et al. (2013) they can be used to evaluate different scenarios and allow the investigation of essential variables that contribute to the UFT phenomenon. However, traditional modeling techniques do not allow us to estimate the variables that affect UFT, since most existing models are based on correlation and regression relationships which, as Pearl and Mackenzie (2018) state, do not tell us the causes and effects that affect the functioning of systems. Therefore, to support better decision-making, it is essential to invest in developing causal models capable of, first, representing the functioning of systems and their cause-and-effect relationships and, second, estimating latent variables present in the urban context. Thus, the circulation of goods and products in consumption centers needs systematic efforts to understand the UFT phenomenon and to produce sustainable planning and operational actions. The lack of balance in UFT planning and operation is reflected in externalities. Economic imbalances create environmentally and, consequently, social imbalances, all cyclicals.

One way to mitigate the externalities of UFT would be through actions that understand the *modus operandi* of this transport and seek to minimize its externalities. In this context, City Logistics (CL) (Taniguchi et al., 2001) has as its guiding principle the development of policies that ensure sustainability to UFT. Therefore, the success of the policy development process is a consequence of the initial effort to understand the problem. Within the process of understanding urban issues, integrated representations of the urban environment are more promising, as they do not seek isolated solutions but rather solutions that address the wants and needs of all the actors that make up the urban environment (Garcia, 2016; Cassiano et al., 2021). In logistics operations, however, transport is also considered a cause of impacts to the urban environment due to being the main responsible for externalities to the environment, propagated through fuel consumption and carbon dioxide (CO_2_) emissions, with road freight transport being considered one of the main contributors to these effects, however, there is not always convergence in the wishes and needs of the actors, which is enhanced by the lack of knowledge of the phenomenon. Brito et al. (2018) showed that in the São Paulo Metropolitan Region, although they represent 5% of the fleet, cargo transport vehicles and buses would be responsible for about half of the local air pollution in terms of nitric oxides (NO) and particulate matter (PM). In addition, logistics operations contribute to the worsening levels of congestion, accidents, noise generation and vibrations observed in urban environments (McKinnon et al., 2010; Oliveira et al., 2019).

Therefore, one of the main challenges of the transportation sector is to combine its activities with the commitment and responsibility for health, welfare, quality of life of the population and environmental preservation. In this sense, it is necessary that freight transport is incorporated in an inclusive way into urban transport planning. This incorporation requires a complete understanding of the object of planning, considering not only the negative externalities of urban freight transport but also its importance in the urban economy and the needs of the parties involved (McKinnon et al., 2015; Oliveira et al., 2019). It arises then, within the academic and professional community, the need for studies and analysis aimed at the behavior of the urban freight transport system (UFC) and its environmental effects, in a dynamic way, as a contribution to urban planning and public policies in cities.

When addressing the effect of the urban environment on the process of pollutant emission and dispersion, it can be assumed that there is an intrinsic relationship between the Activity System (AS), the human needs and desires, and the Transportation System (TS), which supports and organizes vehicular traffic (Frank, 2004; Lopes et al., 2019). The AS is related to aspects such as land use and urban density. Traffic flow, speed, and density are affected by the phenomenon of trip generation, which together with land use and urban density will alter pollutant emission factors (Hong and Goodchild, 2014; Zhang et al., 2018). One way to evaluate vehicle operational performance against urban arrangements arising from activity systems and transportation systems is through the representation of driving cycles, which is a procedure for determining vehicle emissions and is made up of a set of parameters such as speed, acceleration, distance, duration and frequency of starts and stops with the aim of simulating a driving pattern close to reality (Martins, 2005). Such a technique presents relevance for the evaluation of fuel consumption and air emissions, since it depicts the usual behavior of the vehicle in the traffic of a region (Rodríguez et al., 2016). In this way, it is possible to create a driving profile that most closely matches the traffic reality of the region under study (Martins, 2005), thus it can be assumed that the type of road can bring impacts on the driving cycle. The functional classifications are usually listed according to the maximum allowed speed, number of lanes, presence of traffic lights, control devices at intersections, pedestrian crossings, access to neighboring lots, which will have an impact on traffic behavior and, consequently, on fuel consumption and pollutant emissions.

Thus, the central objective of this article is to evaluate the effects of driving style, through the construction of driving cycles, on the CO_2_ emissions from urban freight transport, in the real driving emissions context. In addition, the impacts of the types of roads on the recovered driving cycles will be evaluated, as well as the aspects of land use along the studied roads. To do this, the truck was monitored using Portable Emissions Measurement System (PEMS), Global Positioning System (GPS), exhaust flow meters and On-Board Diagnostic (OBD).

## 2 Materials and methods

The proposed method for the execution of the objectives of this work is organized in the following steps: (i) selection of roads according to functional classification; (ii) definition of periods for experimenting according to traffic conditions; (iii) definition of vehicle type and collection method; (iv) selection of drivers and construction of driving cycles; (v) analysis of CO_2_ emissions; and (vi) correlation of the results obtained with causal factors.

### 2.1 Selection of roads, traffic conditions, and experimental vehicle

**Figure 1:**
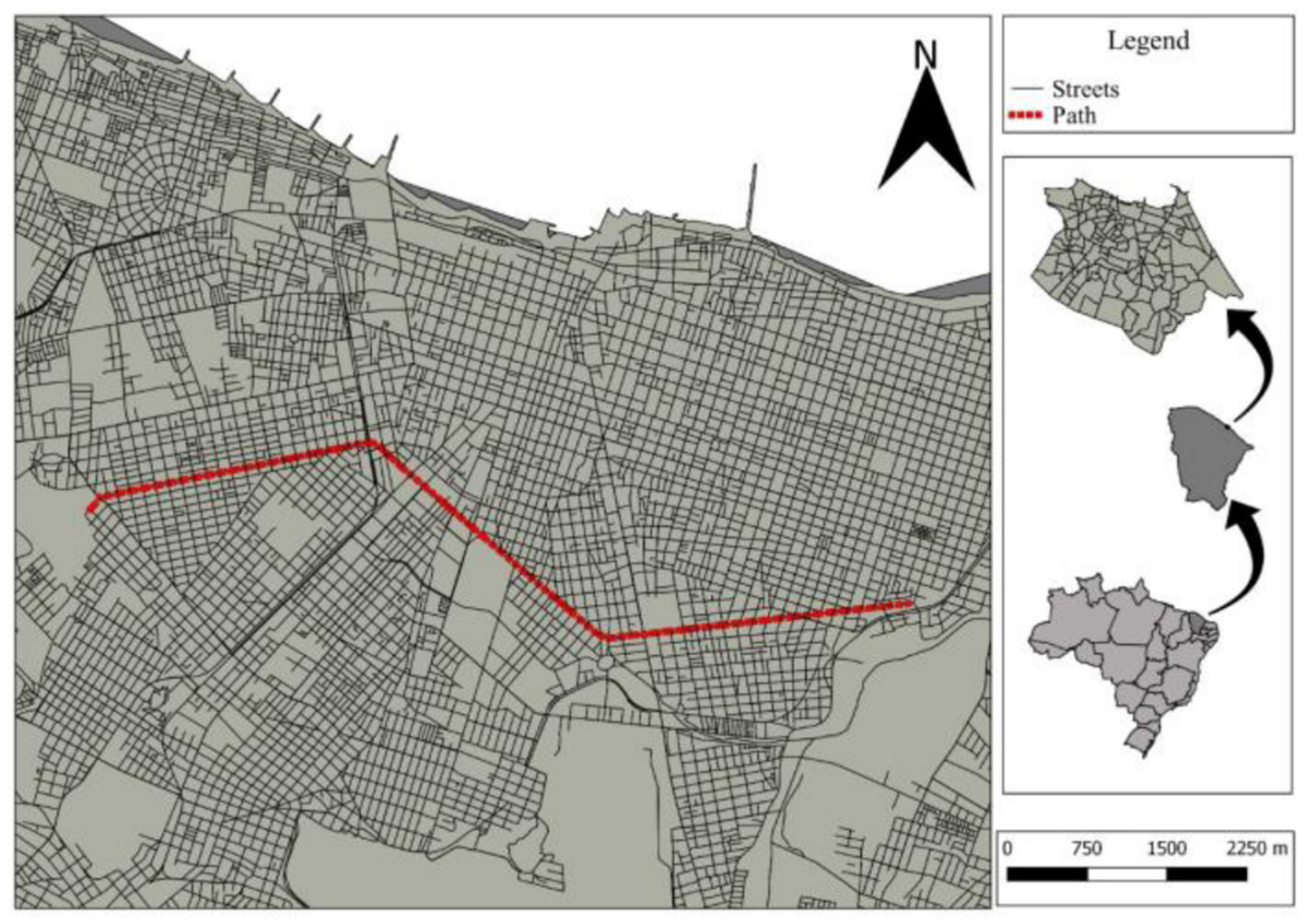
Driving cycle route carried out in Fortaleza city, Ceará, Brazil.

The experiments performed in this work took place in Fortaleza city, Ceará, Brazil. To verify the characteristics of the roads that influence the dynamics of pollutant emissions, the Law of Land Use and Occupancy of Fortaleza was used to determine the region where the experiment would be performed (Fortaleza, 2012). Arterial and collector roads were chosen, in which passenger flows predominate and serve large displacements along the road and connect local roads to arterial roads, respectively. The selection of the roads under study considered the following criteria: same number of lanes, tracks, and presence of a median; same direction and geographical orientation in the city; same road classification; same maximum allowed speed; similar lengths; differences between residential and commercial land use; the presence of commercial activities. The study area consists of a stretch of ~17 km among the three roads and in both directions (West-East and East-West), located in the urban perimeter of Fortaleza city. The route is composed of three ample avenues, classified as a collector, Avenida Jovita Feitosa, and arterial type I, Avenues Treze de Maio and Pontes Vieira. A more residential area surrounds the collector road and the arterial roads are characterized by a higher concentration of commercial businesses, whose traffic volumes between these two roads are similar. Figure 1 shows the complete route, both in the West-East (outbound) and East-West (return) directions. All streets have a maximum allowed speed limit of 60 km/h, and Table 1 presents the selected streets and their characterization according to the criteria listed above.

**Table 1:** Characterization of the selected routes.

**Ways**	**Functional classification**	**Adopted nomenclature**	**No. of tracks**	**Central bed**	**The extension (km)**	**Geographic orientation**
Jovita Feitosa	Collector	C1	2	Yes	2,60	West-East
Jovita Feitosa	Collector	C2	2	Yes	2,60	East-West
Treze de Maio	Arterial I	A1	2	Yes	2,90	West-East
Treze de Maio	Arterial I	A2	2	Yes	2,90	East-West
Pontes Vieira	Arterial I	A3	2	Yes	2,40	West-East
Pontes Vieira	Arterial I	A4	2	Yes	2,40	East-West

The peak loading times of the roads (Peak) were from 6:30 am to 8:30 am, and from 5:30 pm to 7:30 pm, periods characterized by a high number of trips for work and study reasons; and the periods with the lowest flow (Off-Peak) were from 8:30 am to 10:30 am, and from 2:30 pm to 4:30 pm. The most suitable days of the week for traffic studies, according to the literature and confirmed from the analysis of Google Maps^®^ images, are Tuesday, Wednesday, and Thursday, as they have a more homogeneous flow and are less influenced by events such as weekend (Gao, 2007). The period of the year also considered the most suitable for conducting traffic surveys, is the one with the least influence of school holidays, recesses, and winter, when applicable, as they present less variation in flow (Wright et al., 1997). The experiments were performed on Tuesdays, Wednesdays, and Thursdays, considering the school period and disregarding holidays. To reduce the impacts generated by trucks, urban centers have adopted restrictive measures for circulation in certain regions of the city (Brasil, 2012, 2015; Oliveira et al., 2019). In Fortaleza city UFCs have been authorized to operate full time in areas with restrictions on the circulation of larger trucks, making them customary in goods distribution activities in the urban perimeter, and they must meet the limits of length (7.30 m), width (2.20 m) and height (4.40 m), with a maximum total gross weight of up to 10 tons (Fortaleza, 2012). Table 2 shows the characteristics of the UFC adopted in this study.

**Table 2:** Basic characteristics of the UFC used in the study.

**Characteristics**	**UFC information**
Brand/Engine	Foton; Cummins ISF2.8 Diesel
Year/Model	2014/2014
Transmission system	manual 5-speed
Length (m) × Width (m) × Height (m)	4.80 × 1.82 × 2.20
Average weight with trunk (kg)	4,300
Fuel	Diesel, 7% (v/v) biodiesel
Exhaust gas treatment system/Emission standard	EGR/Proconve P7/Euro V
Brand/Engine	Foton; Cummins ISF2.8 Diesel

### 2.2 Selection of drivers and construction of driving cycles

A total of 19 professional truck drivers, aged between 21 and 70, with an average income of 2 minimum wages, drove the vehicle the entire way. The driving data were collected through an On-Board Diagnostic (OBD) device connected to the engine control unit (ECU) and monitored the dynamic parameters of the engine continuously, such as speed, acceleration, engine rpm, inlet airflow in the combustion chamber, revolutions per minute (RPM), and engine load. As for the track parameters, the specific location of the vehicle (latitude, longitude, and altitude) and track inclination are highlighted. All these parameters were obtained at a frequency of 1 Hz. The Vehicle Specific Power (VSP) parameter is a simplified balance of all the forces applied to the vehicle while it is being driven, which gives an estimate of the power per unit mass of the vehicle at each instant, and the operating modes for the construction of the driving cycles were calculated according to Jimenez-Palacios (1998), as shown in Equation 1.


(1) $$\begin{array}{l} VSP\;\left(kW/ton\right)=v^{*}\left(a+g^{*}sin\left(\phi \right)+\text{Ψ}\right)+\zeta^{*}v3 \end{array}$$

Where: v is the speed (m/s); a is the acceleration (m/s^2^); g is the acceleration of gravity, equal to 9.81 m/s^2^; ϕ is the slope of the track; ψ is the rolling resistance coefficient; ζ is the drag coefficient.

### 2.3 Data collection and statistical analysis

All measured and calculated data were organized in Excel and exported to the IBM SPSS 25 statistical package. Each observation obtained was considered as n, which varied according to the travel time for each driver and with a significance of 0.05. First, we checked whether the data followed normality through the parametric test based on the t-student distribution. Since the data collected did not follow a normal distribution, the other tests adopted involved non-parametric methods. For the comparison of pairs of drivers, the Wilcoxon rank sum test was used. For independent samples, either of drivers and/or streets, the Kruskal–Wallis test was used.

**Figure 2:**
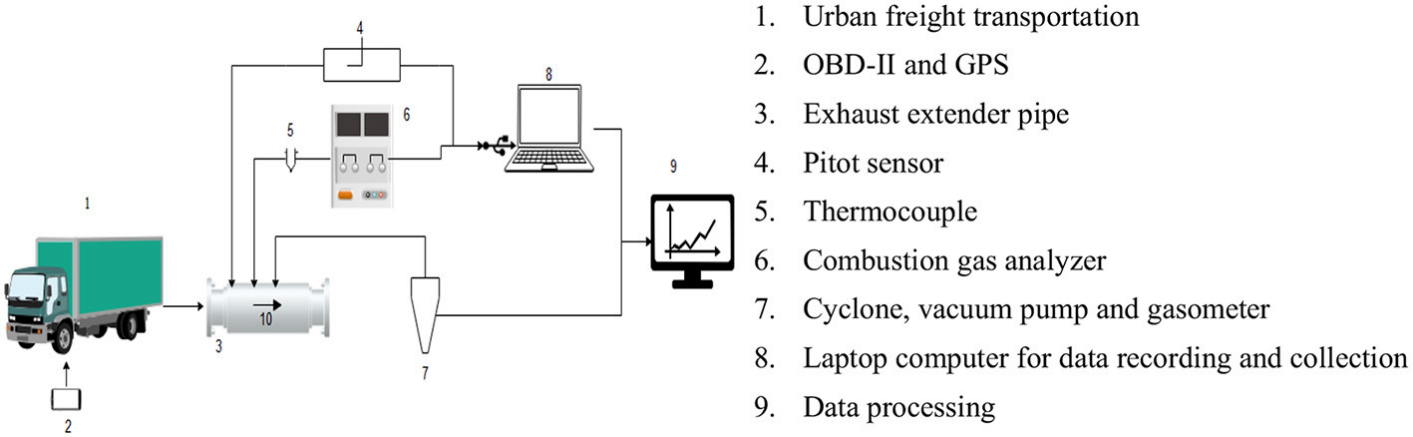
Data collection scheme for vehicular monitoring. Adapted from Cassiano et al. (2016).

To collect emissions, we followed the method described by Cassiano et al. (2016) to obtain real and instantaneous data on pollutant emissions, by Ecil Chemist 400 combustion analyzer, which measures O_2_, CO, NO/NO_x_, SO_2_, NO_2_, C_x_H_y_, CO_2_ and H_2_S, and in this paper, CO_2_ emissions are the target of study, as summarized in Figure 2. Therefore, crossings were made on each roadway in each direction, totaling 17 km and the number of crossings considered two criteria: (i) a minimum of 3 crossings per route; (ii) maximum number of crossings that could be made in each period (peak and off peak), which totaled 20 h of instantaneous data (1 measurement per second) collected, totaling more than 70,000 valid data observations for each of 50 parameters evaluated.

## 3 Results and discussion

Table 3 presents the characteristics of the actual driving cycles of drivers in both directions, West-East and East-West, of Jovita Feitosa (C1 and C2), Treze de Maio (A1 and A2), and Pontes Vieira (A3 and A4) Avenues, in according of Table 1, and FTP-75 and US06 standards for the parameters speed, acceleration, and operating modes (idle, coast, cruise, acceleration, and deceleration).

**Table 3:** Basic characteristics of the UFC used in the study.

	**C1**	**C2**	**A1**	**A2**	**A3**	**A4**	**FTP75**	**US06**
Average speed (km/h)	27.41	25.70	15.76	15.86	14.37	13.22	13.2	29.8
Max speed (km/h)	68.98	55.98	56.99	58.97	58.97	63.00	35.2	49.9
Max acceleration (m/s^2^)	5.275	4.025	1.950	1.940	1.665	1.945	0.570	1.450
Minimum acceleration (m/s^2^)	−3.190	−8.880	−3.335	−3.055	−0.099	−2.640	−0.570	−1.191
Average positive acceleration (m/s^2^)	0.402	0.437	0.444	0.465	0.457	0.465	0.197	0.259
Deceleration (%)	23.0	24.6	40.9	41.2	19.6	17.9	-	-
Idle (%)	13.6	15.1	20.2	19.0	34.9	35.5	-	-
Coast (%)	21.7	21.0	19.3	19.1	18.3	18.9	-	-
Cruise/acceleration (%)	41.7	39.4	19.6	20.8	27.2	27.7	-	-

In Brazil, the driving cycle used to determine emissions from light vehicles and light commercial vehicles is American Federal Test Procedure 75 (FTP-75). The procedures for this test are described in Brazilian Standard NBR 6601 (NBR 6601, 2012). The FTP-75 lasts 1,800 s, reaching a maximum speed of 35.2 km/h and an average speed of 13.2 km/h. In terms of acceleration, the maximum is 0.57 and the average is 0 m/s^2^. In addition to this driving cycle and in view of the driver's aggressiveness, a complementary driving cycle was developed, the FTP US06. This cycle lasts 600 s and reaches a maximum speed of 49.9 km/h and an average speed of 29.8 km/h. During the test, the maximum acceleration is 1.45 m/s^2^ and the average is 0 m/s^2^.

When comparing the actual driving cycles developed in the streets, as shown in Table 3, the differences between the streets whose hierarchical classifications are different are striking, with collector streets having much higher average speeds compared to type I arterial streets (*p*-value 0.012). The comparison between each pair of streets and directions WE-EW were not statistically significant (*p*-value 0.000). Comparing with the standard cycles FTP-75 and US06 it can be seen, in terms of average speed, that there is more influence of the types of streets than of the aggressiveness of the drivers. Also, in Table 3, we can see that the speed parameter is distributed around the median in the collector roads, whose averages were higher concerning the other streets under study. In both directions, the type I arterial roads present higher averages and medians.

**Figure 3:**
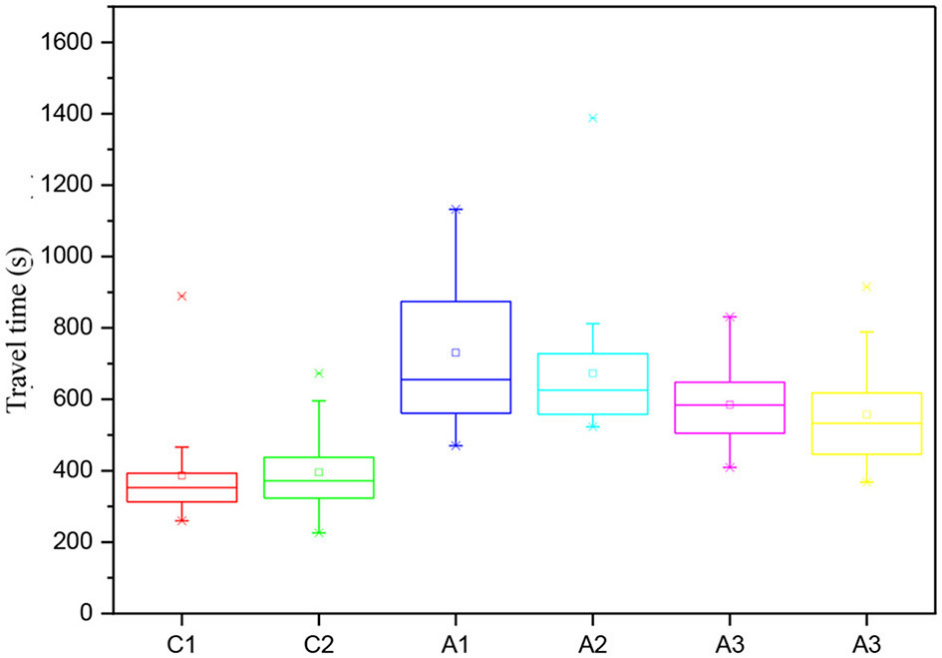
Variation of travel times of collectors and arterials.

**Figure 4:**
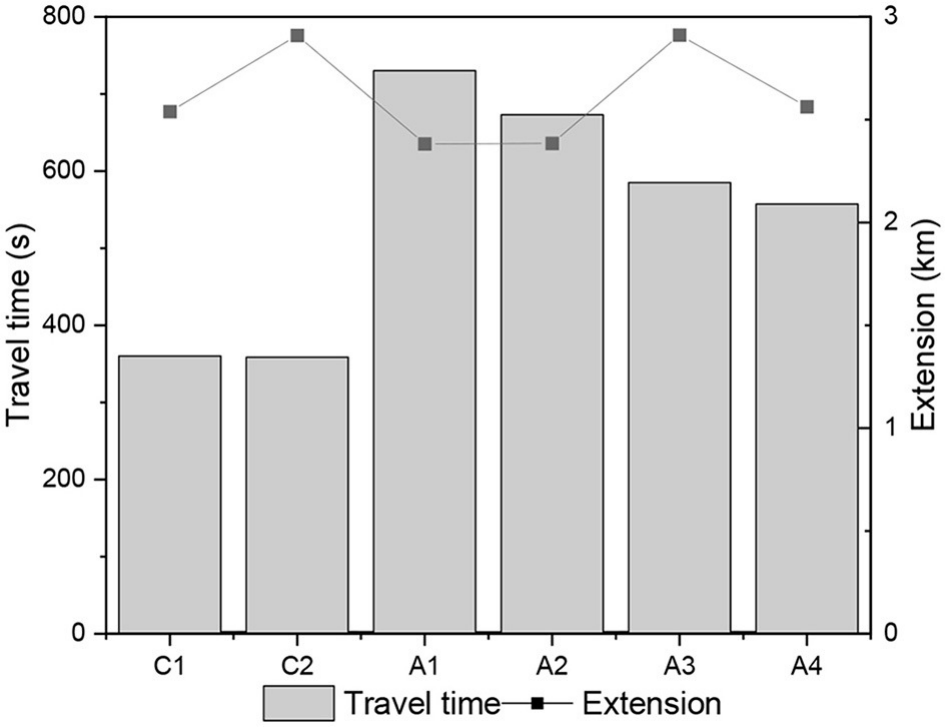
Length of stretches and average travel times in the WE and EW directions of arterials and collector roads.

Regarding the acceleration parameter, the highest amplitudes were observed in the collector roads and the maximum speeds. In general, all accelerations (mean, positive mean, maximum and minimum) of the real driving cycles have larger amplitudes than the standard driving cycles, even the aggressive cycle US06. Linear profiles with averages and medians around zero were observed in all the real driving cycles obtained. Smaller amplitudes for this parameter were observed in both directions of A1 and A2 streets. In general, the variability of the velocities and acceleration parameters were (A1~A2) < (A3~A4) < (C1~C2). Figure 3 shows the variation of observed data of travel times utilizing quartiles (Boxplot). The lowest travel times were observed in the collector streets, followed by streets A3 and A4, and finally, A1 and A2. Except for section A1, the other sections showed normal distribution for the TV parameter, which showed more significant variability for the roads under study. Although the A1 and A2 sections are slightly larger in length, it would not justify such a large difference in travel times, as shown in Figure 4. Thus, it is believed that other local aspects have more influence on travel time.

To search for factors and relationships that better explain these very different emissions, especially between the collector type roadway and the type I arterials. As discussed in Ericsson's (2000) conceptual model and in the scope of testing the hypothesis that local urban aspects can influence the driving mode, we sought more robust information about the land use in each analyzed stretch. Table 4 presents the composition of the neighboring plots for each period of road.

**Table 4:** Land use on collector and arterial avenues.

	**C1**	**C2**	**A1**	**A2**	**A3**	**A4**
Residential (%)	53.06	50.00	46.09	27.38	17.57	7.32
Commercial (%)	28.57	29.90	32.17	53.57	60.81	63.41
Mixed (%)	13.78	18.14	14.78	11.90	13.51	19.51
Others (%)	4.59	1.96	6.96	7.14	8.11	9.76

The sections of the collector roads are composed of more residential land use, exceeding 50%. On the other stretches, arterials the land use (LU) is predominantly commercial and mixed. The commercial LU was believed to interfere more with local traffic due to access to lots, increased local traffic, and even parking on the roadway for both cars and UFTs. However, it is observed that lower speeds are on the collector roads. Still, in the search for factors that better explain these relationships, we also searched the shape of the traffic lights located in Fortaleza city (Table 5).

**Table 5:** Land use on collector and arterial avenues.

	**C1**	**C2**	**A1**	**A2**	**A3**	**A4**
Number of links	24	25	24	26	21	21
Number of traffic lights	8	8	13	13	11	11
Average distance between traffic lights (km)	0.33	0.33	0.22	0.22	0.22	0.22
Average distance between traffic lights on the links	3.00	3.13	1.85	2.00	1.91	1.91

**Figure 5:**
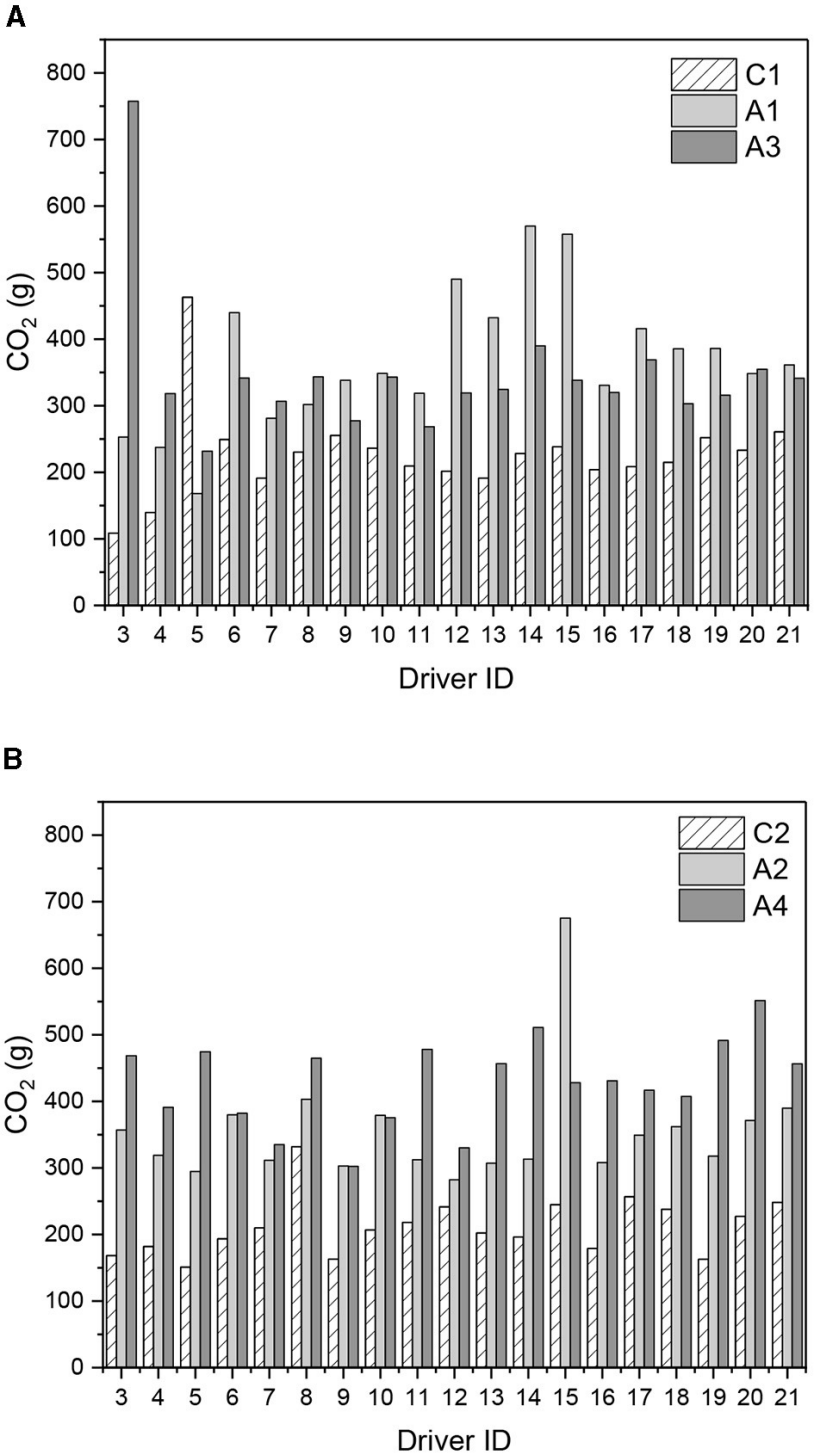
Total CO_2_(g) emissions: in the **(A)** West-East and **(B)** East-West directions.

The number of traffic lights and the reduced distance between them favors the stop and go phenomenon, in which vehicles accelerate and repeatedly stop, making it challenging to develop more constant speeds (cruise control modes), increasing travel times and other impacts on the roads. Thus, it reinforces the need to better plan and organizes traffic, especially in urban centers with greater circulation of people and cargo. When comparing the actual driving cycles developed on the roads, the differences between the streets whose hierarchical classifications are different are notorious, being Jovita Feitosa Avenue, a collector, has average speeds much higher than the other two, Treze de Maio and Pontes Vieira, both arterial type I. Between each pair of streets, directions WE-EW, the differences are not significant; these directions, at times considered, weave a relationship with peak and off peak volumes. Evaluating the standard cycles FTP-75 and US06 and comparing them with the actual driving cycles obtained, it can be seen, in terms of average speed, that there is more influence of the types of streets than of the aggressiveness of the drivers. To evaluate the emissions of pollutants during the experiments, CO_2_ emissions were measured during driving on each stretch and passage. The averages of the paths per stretch are shown in Figure 5.

As Figure 5 shows, there are no significant differences between the West-East and East-West directions for each driver. However, there are differences between roadway types, with the highest CO_2_ emissions on arterial roads, especially on the roadway where speeds were lower and travel times were longer. Again, this reinforces the importance of urban planning, including in terms of air quality and human health.

## 4 Conclusions

Measuring vehicle emissions is relevant not only for vehicle certification purposes, but also, for example, to estimate the impacts of vehicles under real operating conditions. This work aimed to evaluate the effects of driving style, through the construction of driving cycles, on the emission of pollutant gases in urban freight transport. For this, 19 professional freight transport drivers traveled the same route, which included major collector and arterial roads of the city. More than 70,000 observations were obtained for each parameter collected.

For this, the relationship between actual driving cycles and pollutant emissions was obtained with the VSP parameter, which incorporates vehicle, road geometry and driver aspects. The integration of local elements came from the experimental phase in which drivers drove on streets with different urban characteristics regarding land use, number of traffic lights, and traffic volumes at other times of the day. In general, there are significant differences between the actual driving and standard cycles (FTP-75 and US06) in terms of speed, acceleration, and operating modes. Furthermore, it was noticed that there are discrepancies also when comparing roads of different road classifications, such as collector and arterial roads.

The results also showed that local aspects strongly influence pollutant emissions, in terms of CO_2_, both along the roads and in the total pollutants emitted during the tests. For example, one local aspect that had a major impact on emissions was the number of traffic lights, which interferes with the constancy of speeds and the development of the cruise mode of operation, intensifying short term events such as stop and go; the more inconstant, the greater the variability of emissions.

Considering the attributes, vehicular emissions, and operations of urban freight transport, one can estimate the real impact of the sector on air pollution in the region and design better strategies to mitigate these impacts.

## Data availability statement

The original contributions presented in the study are included in the article/supplementary material, further inquiries can be directed to the corresponding author.

## Ethics statement

The studies involving humans were approved by the Federal University of Ceará, Fortaleza, Ethics Committee. The studies were conducted in accordance with the local legislation and institutional requirements. The participants provided their written informed consent to participate in this study. Written informed consent was obtained from the individual(s) for the publication of any potentially identifiable images or data included in this article.

## Author contributions

JA: Conceptualization, Data curation, Formal analysis, Investigation, Methodology, Project administration, Writing – original draft. DC: Data curation, Formal analysis, Methodology, Writing – review & editing. BB: Conceptualization, Data curation, Formal analysis, Methodology, Project administration, Resources, Supervision, Writing – review & editing.
